# Synchronous Low-Grade Appendiceal Mucinous Neoplasm and Neuroendocrine Tumor Presenting as Pseudomyxoma Peritonei: A Case Report

**DOI:** 10.7759/cureus.114456

**Published:** 2026-08-13

**Authors:** Tyler Stevens, Luther Fleury, Jessica Collera, Kelcie Jagodzinski, Ji Fan

**Affiliations:** 1 General Surgery, Hospital Corporation of America (HCA) Healthcare/University of South Florida (USF) Health Morsani College of Medicine Graduate Medical Education (GME), Brandon, USA; 2 General Surgery, Hospital Corporation of America (HCA) Florida Brandon Hospital, Brandon, USA; 3 Gastrointestinal Pathology, Moffitt Cancer Center, Tampa, USA; 4 Gastrointestinal Surgery, Moffitt Cancer Center, Tampa, USA

**Keywords:** appendiceal neuroendocrine tumor, appendix cancer, cytoreductive surgery and hipec, literature review of disease, low-grade mucinous appendiceal neoplasm, pseudomyxoma peritonei, right colectomy, solitary bone plasmacytoma, surgical case reports

## Abstract

Synchronous primary neoplasms of the appendix are exceptionally rare. We report a low-grade appendiceal mucinous neoplasm (LAMN) and a well-differentiated neuroendocrine tumor (NET) arising within the same appendix and presenting as pseudomyxoma peritonei (PMP). A 44-year-old man undergoing staging for a solitary plasmacytoma of the left acetabulum was incidentally found on computed tomography (CT) to have extensive peritoneal mucinosis. Diagnostic laparoscopy confirmed diffuse PMP, and appendectomy revealed two synchronous appendiceal primaries: a perforated LAMN (pT4a pNX) and a 6 mm, grade 1 NET with lymphovascular invasion (pT3 pNX). After multidisciplinary review, the patient completed radiation therapy for the plasmacytoma and subsequently underwent cytoreductive surgery with hyperthermic intraperitoneal chemotherapy (CRS/HIPEC), achieving complete cytoreduction with 33 lymph nodes negative for malignancy. This case highlights the importance of intraoperative suspicion for an appendiceal origin of PMP, the definitive role of histopathology in identifying synchronous pathologies, and the value of coordinated multidisciplinary care when multiple malignancies coexist.

## Introduction

Neoplasms of the vermiform appendix are uncommon, identified in approximately 1% of appendectomy specimens, and are most often discovered incidentally during surgery for presumed appendicitis or on imaging performed for unrelated indications [1,2]. The most common histologic types are epithelial mucinous neoplasms and neuroendocrine tumors (NETs) [3]. Appendiceal NETs are identified in 0.3%-0.9% of appendectomies [4], while low-grade appendiceal mucinous neoplasms (LAMNs) occur in approximately 0.2%-0.3% [5].

LAMNs are characterized by the proliferation of mucin-producing epithelium that may cause cystic dilation of the appendix and, importantly, may rupture and disseminate mucin and neoplastic cells into the peritoneal cavity [6]. This dissemination produces pseudomyxoma peritonei (PMP), a syndrome of progressive gelatinous ascites and mucinous peritoneal implants, classified histologically as acellular mucin, low-grade mucinous carcinoma peritonei, high-grade mucinous carcinoma peritonei, or high-grade disease with signet ring cells [1,6]. Appendiceal NETs are most often located in the distal third of the appendix and are usually discovered incidentally, as they are rarely visualized on preoperative imaging and infrequently cause luminal obstruction [7]. When symptomatic, they most commonly present as acute appendicitis; chronic right lower quadrant pain, a palpable mass, or carcinoid syndrome occurs less frequently [7].

Although LAMN and NET are individually the most common appendiceal neoplasms, their synchronous occurrence as two distinct primary tumors within the same appendix is rare, with an estimated incidence of 2.5 per 1,000 appendectomy specimens [3]. We describe a patient in whom a synchronous LAMN (pT4a) and NET (pT3, grade 1, with lymphovascular invasion) presented as extensive PMP, discovered incidentally during the staging workup of a solitary plasmacytoma. We review the relevant literature and discuss the diagnostic and management considerations raised by this scenario.

## Case presentation

A 44-year-old man on active military duty presented with left hip and buttock pain of approximately one year’s duration. His history was significant for a testicular Leydig cell tumor treated with orchiectomy in 2008, medically managed hypertension, and military burn pit exposure. Radiographs and magnetic resonance imaging (MRI) of the pelvis demonstrated a large, aggressive-appearing lytic lesion of the left posterior acetabulum measuring 7.5 × 5.5 × 3.2 cm. Computed tomography (CT)-guided biopsy confirmed a plasmacytoma composed of atypical plasma cells positive for CD138 and MUM1 with lambda light-chain restriction and a Ki-67 proliferation index of approximately 30%. Bone marrow biopsy showed normocellular marrow with 2% plasma cells and no clonal population by flow cytometry, and positron emission tomography (PET)/CT demonstrated intense fluorodeoxyglucose (FDG) uptake confined to the acetabular lesion (maximum standardized uptake value: 12.6), supporting the diagnosis of solitary plasmacytoma [8]. Because of pain and the risk of pathologic fracture, the patient required a walker with non-weight-bearing precautions for the left leg, and definitive radiation therapy (40-45 Gy) was planned.

Staging contrast-enhanced CT of the chest, abdomen, and pelvis incidentally revealed extensive peritoneal disease, with diffuse peritoneal thickening, omental caking, and mucinous ascites surrounding the liver and spleen, suggestive of PMP. The PET/CT demonstrated the ascites but showed no FDG-avid peritoneal disease, consistent with the low metabolic activity typical of low-grade PMP [9]. The patient was asymptomatic from an abdominal standpoint: he denied abdominal pain, nausea, vomiting, distension, and change in bowel habits, and his weight was stable. On examination, the abdomen was soft, nondistended, and nontender.

He was referred to surgical oncology, and diagnostic laparoscopy with peritoneal fluid aspiration, biopsies, and appendectomy was performed. Intraoperatively, extensive PMP was confirmed, with gelatinous mucin and peritoneal implants in all four quadrants, including the surfaces of the liver and spleen; the Peritoneal Cancer Index (PCI) was 22. The appendix was retrocecal and grossly abnormal, containing a palpable 2 cm tumor with surrounding mucinous deposits. Appendectomy was performed to establish the primary source of the peritoneal disease, and biopsies of representative peritoneal implants, omentum, and the liver surface were obtained. The procedure was well-tolerated with minimal blood loss, and the patient was discharged home the same day.

**Figure 1:**
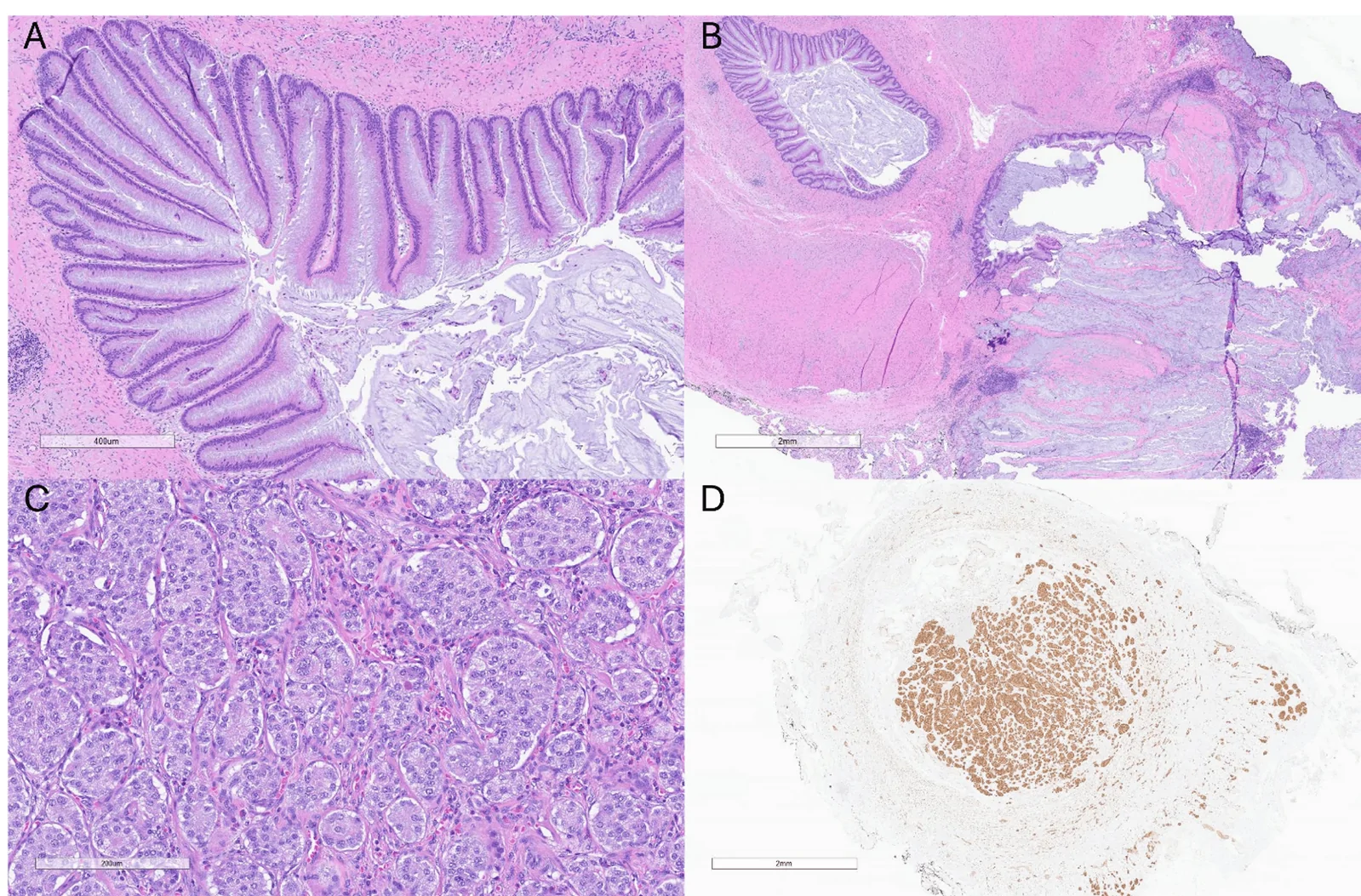
Concurrent low-grade appendiceal mucinous neoplasm (LAMN) and well-differentiated neuroendocrine tumor (WDNET). (A) Replacing the normal appendiceal mucosa is a villous proliferation lined by mucinous epithelial cells, consistent with LAMN. (B) Tumor and acellular mucin can be seen dissecting through the muscularis propria and perforating the wall of the appendix. (C) A concurrent WDNET is present with a nested pattern and cells with speckled (“salt and pepper”) nuclear chromatin. (D) A synaptophysin immunostain is diffusely positive within the NET cells. NET: neuroendocrine tumor

Final pathology identified two distinct synchronous primary neoplasms within the appendix (Figure 1). The first was a grade 1, well-differentiated LAMN with low-grade cytologic atypia, focal perforation, and acellular mucin dissecting through the appendiceal wall to involve the visceral peritoneum; all margins were negative. The second was a 6 mm, well-differentiated, grade 1 NET invading through the muscularis propria into the subserosal adipose tissue and mesoappendix, with lymphovascular invasion present, negative margins, and a Ki-67 index of approximately 1%; tumor cells were positive for synaptophysin, chromogranin A, AE1/AE3, and CAM5.2. The tumors were staged pT4a pNX and pT3 pNX, respectively, per the American Joint Committee on Cancer (AJCC) eighth edition criteria [10,11]. Peritoneal and omental biopsies demonstrated acellular mucin with chronic inflammation and mesothelial hyperplasia, consistent with low-grade PMP, and peritoneal fluid cytology identified rare CDX2- and CK20-positive cells consistent with a disseminated mucinous neoplasm of appendiceal origin, confirming M1a disease.

The postoperative course was uneventful. After the review of the final pathology, multidisciplinary evaluation was initiated to address candidacy for cytoreductive surgery with hyperthermic intraperitoneal chemotherapy (CRS/HIPEC), the sequencing of plasmacytoma radiation relative to major abdominal surgery, the long-term surveillance of the high-risk NET, and genetic counseling in light of multiple primary malignancies at a young age.

Following multidisciplinary discussion, the patient first completed his five-week course of radiation therapy for the acetabular plasmacytoma. Interval colonoscopy revealed three precancerous sessile serrated polyps and two hyperplastic polyps, which were removed, with repeat colonoscopy recommended in three years. Ten days after completing radiation therapy, he underwent cytoreductive surgery, including right hemicolectomy, splenectomy, greater and lesser omentectomy, cholecystectomy, umbilectomy, the resection of the urachus and falciform ligament, the argon beam ablation of tumor deposits, and extensive peritoneal stripping, followed by HIPEC with high-dose mitomycin C. A completeness of cytoreduction score of 0 (CC-0) was achieved, indicating no visible residual disease.

His recovery was complicated by prolonged ileus requiring nasogastric decompression and total parenteral nutrition, a superficial venous thrombosis of the arm, minor superficial wound dehiscence, and small bilateral pleural effusions. He received supportive care and postsplenectomy vaccinations and was discharged with home health services.

Compared with the index pathology, final pathology from the cytoreduction remained consistent with the known LAMN, demonstrating acellular mucin deposits on nearly all resected surfaces, including the gallbladder serosa, liver capsule, and multiple peritoneal surfaces; the distal colonic margin also contained an acellular mucin deposit. The most significant new finding was a focus of well-differentiated mucinous adenocarcinoma involving the splenic capsule (pM1b). All 33 lymph nodes examined were negative for malignancy (0/33), a favorable prognostic finding.

## Discussion

This case is notable for the incidental discovery of extensive PMP during the staging of a solitary plasmacytoma and for the identification of two distinct synchronous appendiceal primaries, which included a perforated pT4a LAMN and a pT3 and a grade 1 NET with lymphovascular invasion in a patient with a prior testicular Leydig cell tumor. Synchronous LAMN and NET within the same appendix have been documented only in isolated case reports and small series [1-3,12]. Such synchronous tumors are best characterized as “collision” tumors, which are described as two independent neoplastic processes arising coincidentally within the same organ rather than composite tumors of shared cellular origin [1]. The distinct mucinous epithelial and neuroendocrine histologies observed here support a collision phenomenon. Table 1 summarizes reported cases of synchronous appendiceal LAMN and NET. The present case is distinguished from prior reports by the coexistence of a third active malignancy and by the high-risk features of the NET despite its small size. Of the reported cases, only that of Hajjar et al. similarly presented with peritoneal dissemination [1].

**Table 1:** Summary of selected literature reports on synchronous appendiceal LAMN and NET. LAMN, low-grade appendiceal mucinous neoplasm; NET, neuroendocrine tumor; RLQ, right lower quadrant; NR, not reported; LGMA, low-grade mucinous adenocarcinoma; RHC, right hemicolectomy; CRS/HIPEC, cytoreductive surgery with hyperthermic intraperitoneal chemotherapy; M, male; F, female

Reference/Year	Age (Years)/Sex	Presentation	LAMN Details	NET Details	Management
Sugarbaker et al., 2020 [3]	39/F	RLQ pain	LAMN (2.3 cm), PT3N0M1a	NET G1, 1.7 cm, pT1bN0	RHC and CRS/HIPEC
Sugarbaker et al., 2020 [3]	32/M	Incidental during hernia surgery	LAMN (size NR), PT3N0M1a	NET G1, 0.5 cm, G2, pT2N1MX	RHC and CRS/HIPEC
Das et al., 2017 [2]	67/F	Appendicitis symptoms	LAMN, proximal appendix	NET G1, 1.1 cm, tip	Appendectomy, colonoscopy, planned RHC (outcome NR)
Villa et al., 2021 [12]	31/F	Abdominal pain, dysuria (appendicitis)	LAMN Tis, base, positive margin	NET G1, pT3 (mesoappendiceal invasion), Ki-67 <1%	Appendectomy, laparoscopicRHC
Hajjar et al., 2019 [1]	50/M	Perforated appendicitis, abscess	LAMN progressing to LGMA, 5.5 cm, perforated, positive margin	NET G1, 1.6 cm, muscularis/minimal mesoappendiceal invasion	Appendectomy, RHC and CRS (peritoneal metastases)

The etiology of synchronous appendiceal tumors remains speculative; proposed explanations include prolonged exposure to shared intraluminal carcinogens, distinct carcinogenic pathways acting simultaneously, and stochastic coincidence [3]. In this patient, the history of military burn pit exposure raises the possibility of an environmental contribution, and the occurrence of multiple primary malignancies at a relatively young age supports genetic counseling to evaluate for an underlying predisposition syndrome.

Appendiceal neoplasms most often present with symptoms mimicking acute appendicitis or are discovered incidentally [1,2], and PMP itself is frequently insidious, remaining asymptomatic or producing only vague symptoms such as increasing abdominal girth or hernia [9]. In the present case, the patient had no abdominal symptoms despite extensive four-quadrant disease. Preoperative CT identified the peritoneal disease but not the appendiceal tumors, and PET/CT was non-avid, consistent with low-grade histology [3,9]. Diagnostic laparoscopy was essential, allowing the direct assessment of disease extent (PCI of 22), the identification of the abnormal appendix, and tissue acquisition; the definitive characterization of both neoplasms and the confirmation of peritoneal dissemination required detailed histopathologic and immunohistochemical analysis.

The standard of care with curative intent for eligible patients with PMP arising from LAMN is maximal cytoreductive surgery combined with HIPEC [13]. The goal of CRS is the macroscopic removal of all visible disease (CC-0) or residual nodules <2.5 mm (CC-1), with HIPEC (commonly mitomycin C or oxaliplatin-based) targeting residual microscopic disease [9]. Reported five- and 10-year overall survival for low-grade PMP after complete cytoreduction exceeds 75% and 60%, respectively [9]. Because CRS/HIPEC carries substantial morbidity, rigorous patient selection is required, incorporating performance status, physiologic reserve, the extent of peritoneal disease, the exclusion of extra-abdominal metastases, and the predicted feasibility of complete cytoreduction [14]; a high PCI is not an absolute contraindication in low-grade disease if complete cytoreduction is achievable [14]. In this patient, young age was favorable, but the active plasmacytoma requiring radiation, impaired mobility affecting performance status, and four-quadrant disease with a PCI of 22 required careful deliberation. Although surveillance may be considered for select non-perforated LAMNs, the presence of extensive PMP made CRS/HIPEC the standard recommendation [15]. After shared decision-making, radiation therapy was completed first while abdominal workup proceeded, a sequence made feasible by the indolent biology of low-grade PMP and the operational capabilities of a high-volume center.

The synchronous NET added further complexity. Although small (6 mm) and low grade (G1, Ki-67 1%), it demonstrated two adverse features: subserosal invasion (pT3) and lymphovascular invasion [11]. The optimal surgical management of appendiceal NETs <2 cm with high-risk features remains controversial [16]. Simple appendectomy is curative for most tumors <1 cm without adverse features, whereas right hemicolectomy for regional lymphadenectomy is recommended for tumors >2 cm [11]. For smaller tumors with risk factors such as lymphovascular invasion, mesoappendiceal invasion >3 mm, positive margins, base location, or higher grade, the European Neuroendocrine Tumor Society (ENETS) and North American Neuroendocrine Tumor Society (NANETS) suggest considering right hemicolectomy, whereas the National Comprehensive Cancer Network (NCCN) guidance has been variably interpreted as permitting appendectomy alone (Table 2) [17,18]. Recent data suggest that lymphovascular invasion may be a stronger predictor of nodal metastasis than the depth of invasion for tumors <2 cm [19]. In this case, the right hemicolectomy performed as a component of cytoreduction provided the regional lymphadenectomy indicated for the high-risk NET, and all 33 nodes were negative. Long-term surveillance for NET recurrence, including periodic imaging and potentially serum chromogranin A, remains warranted [19].

**Table 2:** Comparison of selected guideline considerations for appendiceal NETs <2 cm. *Risk factors include lymphovascular invasion, mesoappendiceal invasion >3 mm, R1 resection, base location, and grade 2-3 histology. NCCN, National Comprehensive Cancer Network; ENETS, European Neuroendocrine Tumor Society; NANETS, North American Neuroendocrine Tumor Society; RHC, right hemicolectomy; NETs, neuroendocrine tumors

Feature/Guideline Body	NCCN (Variable Interpretations)	ENETS	NANETS
Size <1 cm, no risk factors	Appendectomy	Appendectomy	Appendectomy
Size 1-2 cm, no risk factors	Appendectomy	Appendectomy	Appendectomy
Size 1-2 cm, with risk factors*	Appendectomy (potentially)	Consider/discuss RHC	Consider/discuss RHC
Size <1 cm, with risk factors*	Appendectomy (potentially)	Consider/discuss RHC	Consider/discuss RHC

Finally, this case illustrates the central role of multidisciplinary coordination when multiple malignancies coexist [12]. The principal challenge was treatment sequencing (balancing radiation for the symptomatic plasmacytoma against the timely definitive treatment of the PMP) so that the treatment of one malignancy did not prohibitively increase the risk posed by another.

This report is limited by its description of a single case, and available follow-up is confined to the early postoperative period; long-term outcomes regarding PMP control, NET recurrence, and plasmacytoma response remain unknown.

## Conclusions

We report an exceptionally rare case of synchronous LAMN (pT4a) and grade 1 NET (pT3, with lymphovascular invasion) within the same appendix, presenting as extensive PMP discovered incidentally during the workup of a solitary plasmacytoma. This case underscores the potential for the incidental discovery of significant pathology in asymptomatic patients, the limitations of preoperative imaging and the definitive role of histopathology in complex appendiceal disease, the ongoing debate regarding appendectomy versus right hemicolectomy for small appendiceal NETs with high-risk features, and the importance of integrated multidisciplinary care in sequencing treatment for concurrent malignancies.
